# Maternal thyroid function and offspring birth anthropometrics in women with polycystic ovary syndrome

**DOI:** 10.3389/fendo.2024.1388473

**Published:** 2024-05-29

**Authors:** Anastasia Trouva, Michael Alvarsson, Jan Calissendorff, Bjørn Olav Åsvold, Dorina Ujvari, Angelica Lindén Hirschberg, Eszter Vanky

**Affiliations:** ^1^ Department of Molecular Medicine and Surgery, Karolinska Institutet, Stockholm, Sweden; ^2^ HUNT Center for Molecular and Clinical Epidemiology, Department of Public Health and Nursing, NTNU, Norwegian University of Science and Technology, Trondheim, Norway; ^3^ HUNT Research Center, Department of Public Health and Nursing, NTNU, Norwegian University of Science and Technology, Levanger, Norway; ^4^ Department of Endocrinology, Clinic of Medicine, St. Olavs Hospital, Trondheim University Hospital, Trondheim, Norway; ^5^ Department of Women’s and Children’s Health, Karolinska Institutet, Stockholm, Sweden; ^6^ Department of Microbiology, Tumor and Cell Biology, National Pandemic Centre, Centre for Translational Microbiome Research, Karolinska Institutet, Solna, Sweden; ^7^ Department of Gynecology and Reproductive Medicine, Karolinska University Hospital, Stockholm, Sweden; ^8^ Department of Clinical and Molecular Medicine, Faculty of Medicine and Health Sciences, Norwegian University of Science and Technology, Trondheim, Norway; ^9^ Department of Obstetrics and Gynecology, St. Olavs Hospital, Trondheim University Hospital, Trondheim, Norway

**Keywords:** thyroid function, pregnancy, birthweight, birth length, birth head circumference, PCOS

## Abstract

**Objectives:**

Polycystic ovary syndrome (PCOS) and thyroid disorders have both been linked to adverse pregnancy and neonatal outcomes. Even small variations in thyroid function within the normal range may influence fetal growth. Our aim was to investigate whether maternal thyroid function is associated with newborn anthropometrics in PCOS and explore the potential modifying effect of metformin.

**Methods:**

*Post-hoc* analyses of two RCTs in which pregnant women with PCOS were randomized to metformin or placebo, from first trimester to delivery. Maternal serum levels of thyroid stimulating hormone (TSH) and free thyroxine (fT4) were measured at gestational weeks (gw) 5–12, 19, 32 and 36 in 309 singleton pregnancies. The mean z-scores of birthweight, birth length, and head circumference were estimated in the offspring. Associations of maternal thyroid parameters with offspring anthropometrics and the outcomes large for gestational age (LGA) and small for gestational age (SGA) were studied using linear and logistic regression models, with adjustment for body mass index (BMI) when relevant.

**Results:**

Maternal fT4 at baseline was negatively associated with birth length (b= -0.09, p=0.048). Furthermore, ΔfT4 during pregnancy correlated positively to z-score of both birth weight and length (b=0.10, p=0.017 and b=0.10, p=0.047 respectively), independently of treatment group. TSH at baseline and gw19 was inversely associated with LGA (OR 0.47, p=0.012 and OR 0.58, p=0.042), while ΔTSH was positively associated with LGA (OR 1.99, p=0.023). There were inverse associations between TSH at baseline and SGA (OR 0.32, p=0.005) and between ΔfT4 and SGA (OR 0.59, p=0.005) in the metformin group only. There were no associations between maternal thyroid function and head circumference of the newborns.

**Conclusion:**

In women with PCOS, a higher maternal fT4 in early pregnancy and a greater decrease in fT4 during pregnancy was associated with a lower offspring birthweight and shorter birth length. Higher TSH by mid-gestation and smaller increase in TSH during pregnancy was associated with less risk of LGA. Subclinical variations in maternal thyroid function might play a role for birth anthropometrics of PCOS offspring.

## Introduction

**Figure 1:**
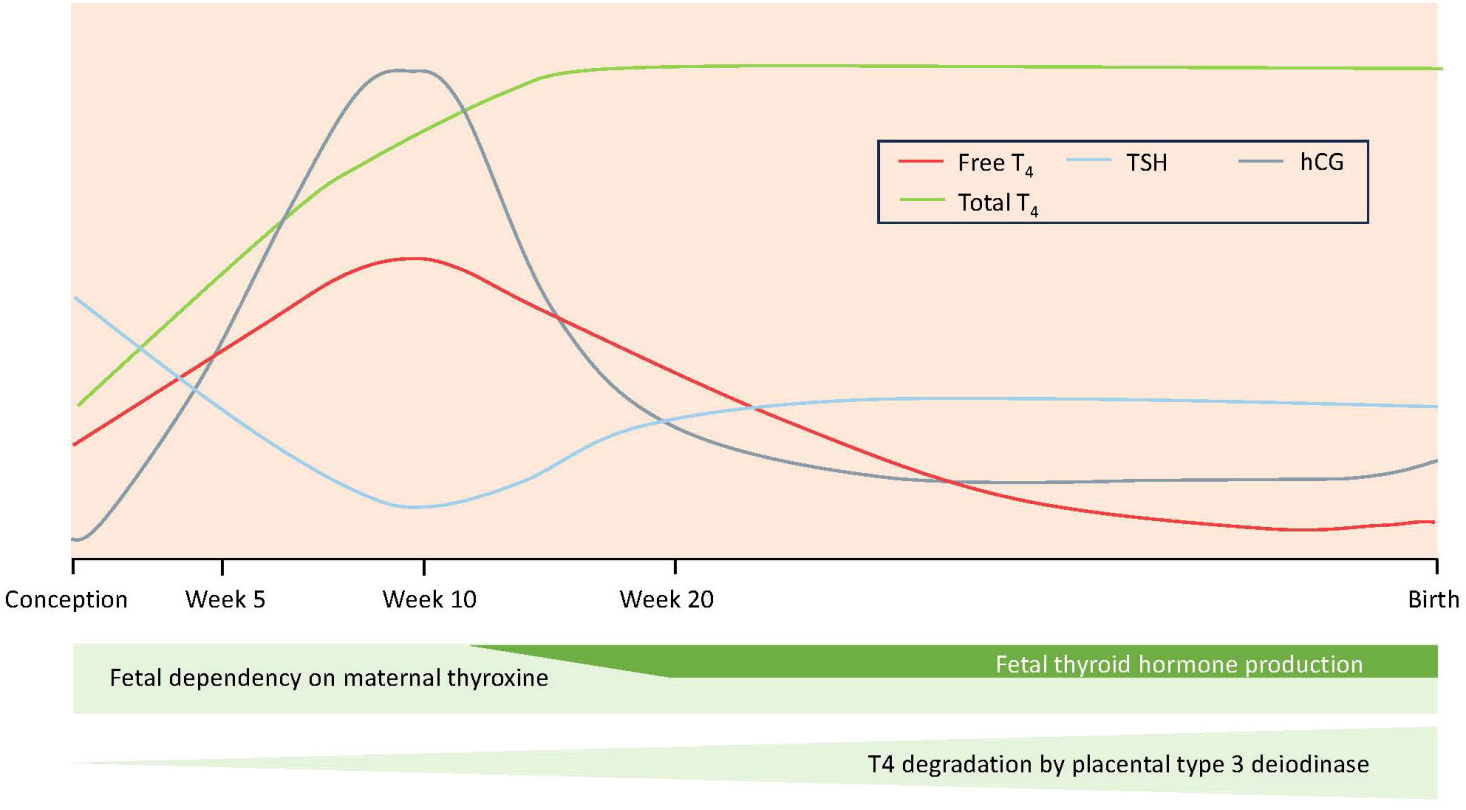
Hormonal changes during a normal pregnancy.

Thyroid hormone availability during pregnancy is crucial for fetal development and maturation (1). In normal pregnancy, increasing concentrations of human chorionic gonadotropin in the first trimester induce a lowering of thyroid stimulating hormone (TSH) concentration mirrored by a transient increase in free thyroxine (fT4). In the 2^nd^ and 3^rd^ trimester, TSH concentration gradually returns to normal and fT4 falls (as thyroid hormone binding globulin (TBG) concentration increases) and may be below the limit of non-pregnant reference range, as placental deiodinase degrades maternal thyroid hormone (**Figure 1**). Overt maternal hypothyroidism (OH) is associated with pregnancy loss, increased risk for premature birth, low birthweight (2), as well as low Apgar score and admission to the neonatal intensive care unit (NICU) (3, 4). Furthermore, thyroid autoimmunity per se has been associated with pregnancy complications and adverse neonatal outcomes (5, 6). Even a mild maternal impairment of the thyroid function may pose an elevated risk for pregnancy and neonatal complications compared to euthyroid women, although the results in these cases are less conclusive. Thus, subclinical hypothyroidism and isolated hypothyroxinemia have in some studies been linked to intrauterine growth restriction, low birthweight and increased risk for small for gestational age (SGA) neonates, smaller head circumference and shorter birth length (7–10), whereas others have not found such associations (11).

Apart from the effect of maternal hypothyroidism on neonatal outcomes, studies also support that thyroid status of the mother within the normal range could influence birth characteristics of the offspring. Most studies report inverse associations between first trimester maternal fT4 and birthweight (12–16), and the highest fT4 levels within the normal range in early pregnancy have even been associated with an increased risk of SGA (14). Furthermore, a longitudinal study of maternal thyroid status showed that an larger increase in TSH between the first and third trimester was associated with lower birthweight (17).

Polycystic ovary syndrome (PCOS) is another common condition among women of reproductive age, with a prevalence around 10–15% depending on the criteria used (18). The syndrome is characterized by oligo- or anovulation, hyperandrogenism (clinical and/or biochemical) and polycystic ovaries (19). Insulin resistance plays a pivotal role in the pathogenesis of the syndrome and has a prevalence of up to 64% among women with PCOS (20). Furthermore, there is an association between PCOS and thyroid disease (21, 22). As thyroid dysfunction, PCOS is linked to adverse pregnancy and neonatal outcomes. According to the newly published International evidence-based guideline for the assessment and management of PCOS, pregnant women with the syndrome have an increased risk for higher gestational weight gain, miscarriage, gestational diabetes, preeclampsia, intrauterine growth restriction, small for gestational age and low birth weight babies, preterm delivery and caesarean section (23).

Metformin treatment during pregnancy in women with PCOS has demonstrated a significant reduction of late miscarriages and preterm births (24). Regarding the thyroid function, metformin treatment is associated with a smaller decrease in fT4 compared with placebo during pregnancy, while TSH is not affected (25). Offspring anthropometrics are also altered by metformin, resulting in larger head size in offspring of PCOS without affecting birthweight and length (26). Whether thyroid hormone status of mothers with PCOS influences offspring anthropometry and neonatal outcomes has not been previously studied, nor a potential interaction between thyroid hormone status and metformin.

The present study is a *post-hoc* analysis of two randomized controlled trials (RCTs) performed to study the effect of metformin on pregnancy complications in women with PCOS (27, 28). We aimed to investigate whether maternal thyroid function (TSH and fT4) assessed longitudinally during pregnancy is associated with newborn anthropometrics and large for gestational age (LGA)/SGA outcomes, and to explore the potential modifying effect of metformin.

## Materials and methods

### Study design

The present study is a *post-hoc* analysis of two randomized, controlled, double-blinded studies with similar protocols; the pilot and the PregMet study (27, 28). In both the pilot and the PregMet studies, pregnant women having PCOS were randomized to either metformin or placebo to investigate the potential of metformin to prevent pregnancy complications. Women were treated from inclusion in the first trimester to delivery with a metformin dose of 1700 mg (pilot) or 2000 mg (PregMet), or placebo. The original studies are described in detail in the **Supplementary Material**.

### Study population

**Figure 2:**
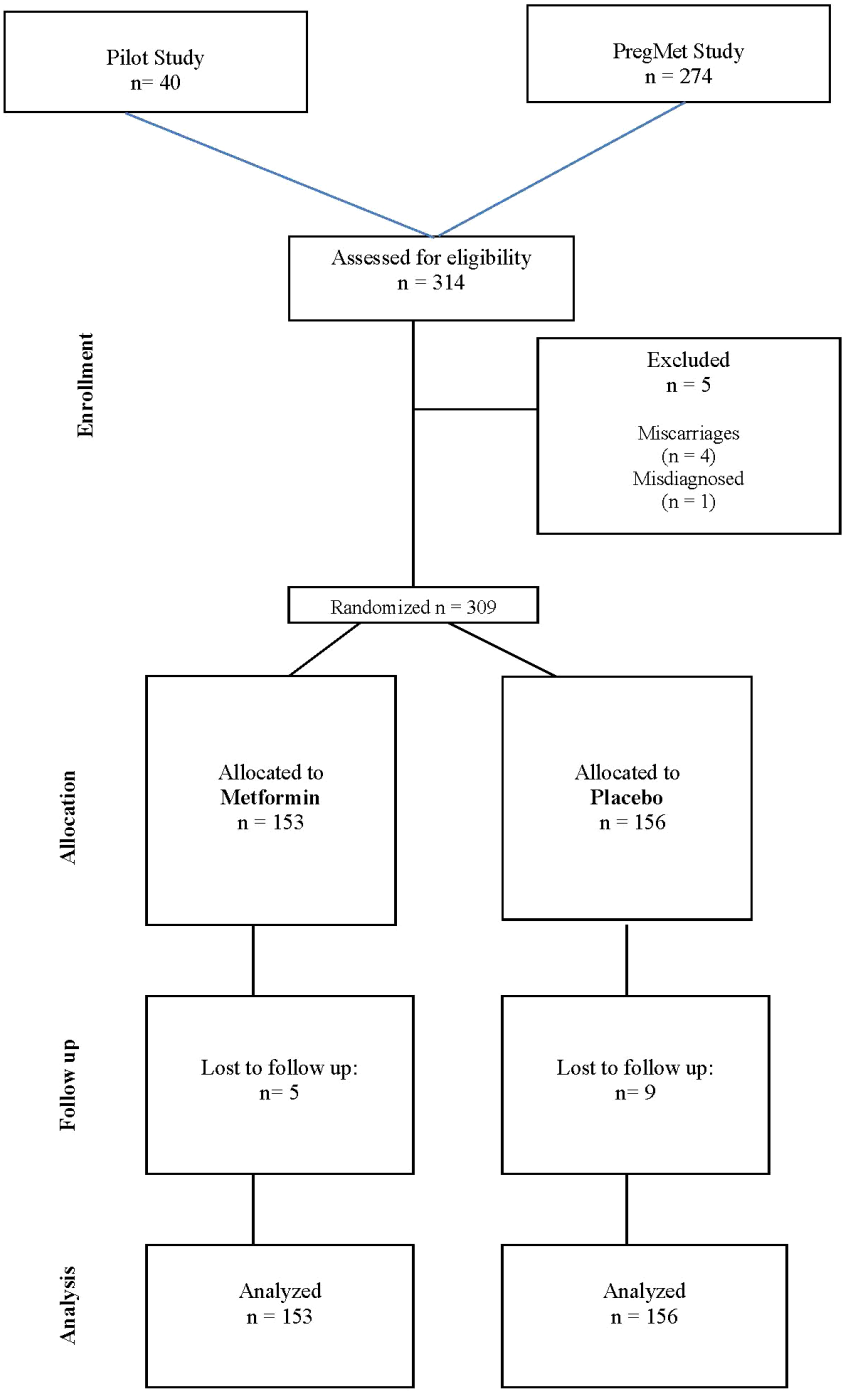
Flowchart of the study design.

The flowchart of eligible women, exclusions, and the final cohort of randomized participants is shown in **Figure 2**. 25 participants participated during two pregnancies, i. e. first pregnancy in the pilot and second in the PregMet study (n=8) or twice in the PregMet study (n=17). Both pregnancies of these participants were included in our analyses. Out of 314 participations with singleton pregnancies, there were 4 miscarriages and one misdiagnosed participant (proved to have congenital adrenal hyperplasia instead of PCOS) who were excluded. In total, data from 309 singleton pregnancies were included in our analyses, of which 153 were randomized to metformin and 156 randomized to placebo. Out of these, 5 in the metformin group and 9 in the placebo group dropped out at some point during the study, however these participants did not ask for withdrawal of their informed consent; therefore, collected data (maternal and offspring) from all randomized participants were included in the present analyses, according to the intention-to-treat principle. Blood samples were collected at inclusion, gw 19, 32 and 36. All blood samples were stored at -70°C.

### Laboratory analysis

TSH and fT4 were measured in samples drawn at inclusion and at gestational week (gw) 19, 32 and 36. For analysis of thyroid peroxidase antibodies (TPO-ab), we used blood samples collected at inclusion and gw 36.

Serum levels of TSH, fT4 and TPO-ab were analyzed by chemiluminescent enzyme immunometric assays using commercial kits procured from Siemens Medical Solutions Diagnostics (fT4 and TPO-ab) and Diagnostic Products Corporation (TSH) (Immulite^®^, Los Angeles, CA). TPO-ab was considered positive if the value was ≥ 35 IU/mL Respective detection limits (and intra-assay and inter-assay coefficients of variation) were as follows: for TSH 0.004 mIU/L (5.0% and 9.9%), for fT4 3.6 pmol/L (4.5% and 5.5%) and for TPO-ab 7 IU/mL (4.3% and 10.5%). The method-specific reference range provided by the manufacturer was 0.4 – 4 mIU/L for TSH and 11.5 – 22.7 pmol/L for fT4.

### Offspring anthropometrics

Head circumference (HC), length, and body weight of the offspring were measured by midwifes and nurse assistants immediately after delivery. For HC, length, and weight at birth, gestational age– and sex-adjusted *z*-scores were calculated based on Niklasson’s standard values from a large Swedish population (29). The standard is based on singleton fetuses without chromosome abnormalities or major birth defects. The *z*-scores express the deviation between observed values and the standard population mean adjusted for sex and gestational age at birth.

### Definition of adverse growth birth outcomes

LGA was defined as birth weight > 90^th^ percentile for gestational age and SGA was defined as birth weight < 10^th^ percentile for gestational age.

### Statistical analyses

Continuous data are summarized as mean ± standard deviation (SD) and categorical data are presented as counts and percentages. In addition, z-scores of birth length, weight and head circumference are presented with 95% confidence interval (CI). Descriptive statistics for TSH and fT4 are presented with median and interquartile range. Comparisons between the treatments were performed using Students t-test (one analysis only with separate variance estimates), Mann-Whitney U test, Chi-square test and Fisher’s exact test. Relationships between the continuous dependent variables z-score for birth weight, length, and head circumference and one in turn of the independent variables TSH, fT4 (at baseline, gw19, gw32 and gw36), change in TSH and fT4 during the pregnancy were analyzed with linear regression. The relationships between the independent variables and the outcomes SGA and LGA (yes/no) (categorical variables) were analyzed with logistic regression to estimate odds ratios (ORs) and 95% confidence intervals (CIs). The treatment factor was included in the regression models. To study whether the relationship between the z-score variables and TSH, fT4 as well as the change in TSH and fT4 was dependent on the treatment, the interaction with treatment was included. In case of no statistical significance, the interaction term was excluded. The residuals were investigated by Cook’s distance. Observations with high Cook’s distance (observations that are far from the “cluster”), were excluded and a new regression analysis was performed. Models with reasonable residual distributions were considered to constitute the final solutions. We considered adjustments of the results of the linear and logistic regression for age and BMI at inclusion, as these parameters could affect the outcomes. However, only BMI was found to be significantly associated to z-score birthweight and head circumference of all the outcomes studied and adjustments were performed accordingly, and we present the adjusted data.

Fifteen women (8 in the placebo group and 7 in the metformin group) with hypothyroidism diagnosis prior to inclusion and treated with levothyroxine, did not differ significantly from the other women, regarding the outcome variables. Thus, these participants were included in our analyses.

P-values < 0.05 were considered as statistically significant.

Statistical calculations were performed using Statistica 14.0 (TIBCO Software Inc.).

## Results

Maternal baseline demographics and clinical parameters were comparable between the metformin and the placebo groups (**Table 1**). As previously reported, TSH increased and fT4 decreased as expected during pregnancy in women with PCOS, whereas metformin treatment was associated with a less decrease in fT4 compared to placebo, while TSH was not affected by metformin (25) (**Supplementary Table S1**).

**Table 1:** Baseline characteristics of women with PCOS at inclusion, first trimester of pregnancy.

	Total (n=309)	Metformin (n=153)	Placebo (n=156)
Age (years)	29.3 ± 4.4	29.5 ± 4.4	29 ± 4.3
Height (cm)	167.5 ± 5.6	167.3 ± 5.8	167.7 ± 5.3
Weight (kg)	81.5 ± 19.5	83.5 ± 20.2	79.4 ± 18.5
BMI (kg/m^2^)	29.2 ± 7.2	29.8 ± 7.0	28.5 ± 7.4
Systolic BP (mm Hg)	118 ± 12	119 ± 12	117 ± 12
Diastolic BP (mm Hg)	73 ± 9	74 ± 9	72 ± 10
Parity n (%)			
P= 0	176 (57)	89 (58)	87 (56)
P≥ 1	133 (43)	64 (42)	69 (44)
Smoking n (%)	22 (8)	14 (10)	8 (6)
Caucasian n (%)	302 (98)	147 (97)	155 (99)
Gestational age at inclusion (days)	72.7 ± 13.8	72.7 ± 14.0	72.8 ± 13.6
Hypothyroidism prior to inclusion n (%)	15 (5.6)	7 (5.2)	8 (5.9)
TPO-ab (+) n (%)	20 (7)	10 (7)	10 (7)
PCOS phenotype			
A (HA+OΑ+PCOM) n (%)	161 (60)	79 (59)	82 (61)
B (HA+OΑ) n (%)	11 (4)	7 (5)	4 (3)
C (HA+PCOM) n (%)	27 (10)	15 (11)	12 (9)
D (OΑ+PCOM) n (%)	71 (26)	34 (25)	37 (27)

BMI, Body Mass Index; BP, blood pressure; TPO-ab, thyroid peroxidase antibodies; PCOS, polycystic ovary syndrome; HA, hyperandrogenism; OA, oligo-/anovulation; PCOM, polycystic ovarian morphology.

Continuous variables are presented as mean ± SD and categorical variables as number (%) of participants.

None of the comparisons between the groups showed statistically significant difference (p>0.05).

**Figure 3:**
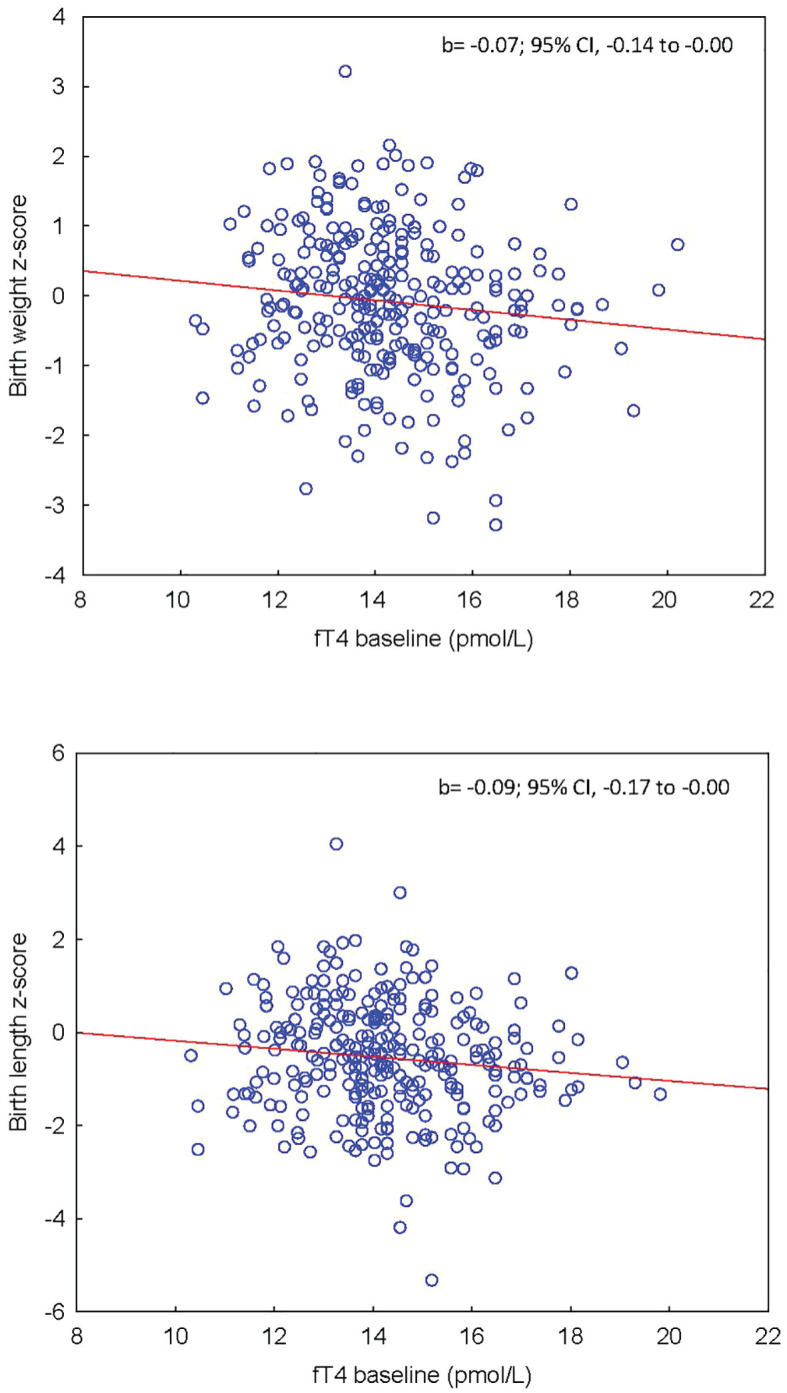
(Upper panel) Association between maternal fT4 at baseline and offspring’s z-score birthweight adjusted for BMI. (Lower panel) Association between maternal fT4 at baseline and offspring’s z-score birth length.

**Figure 4:**
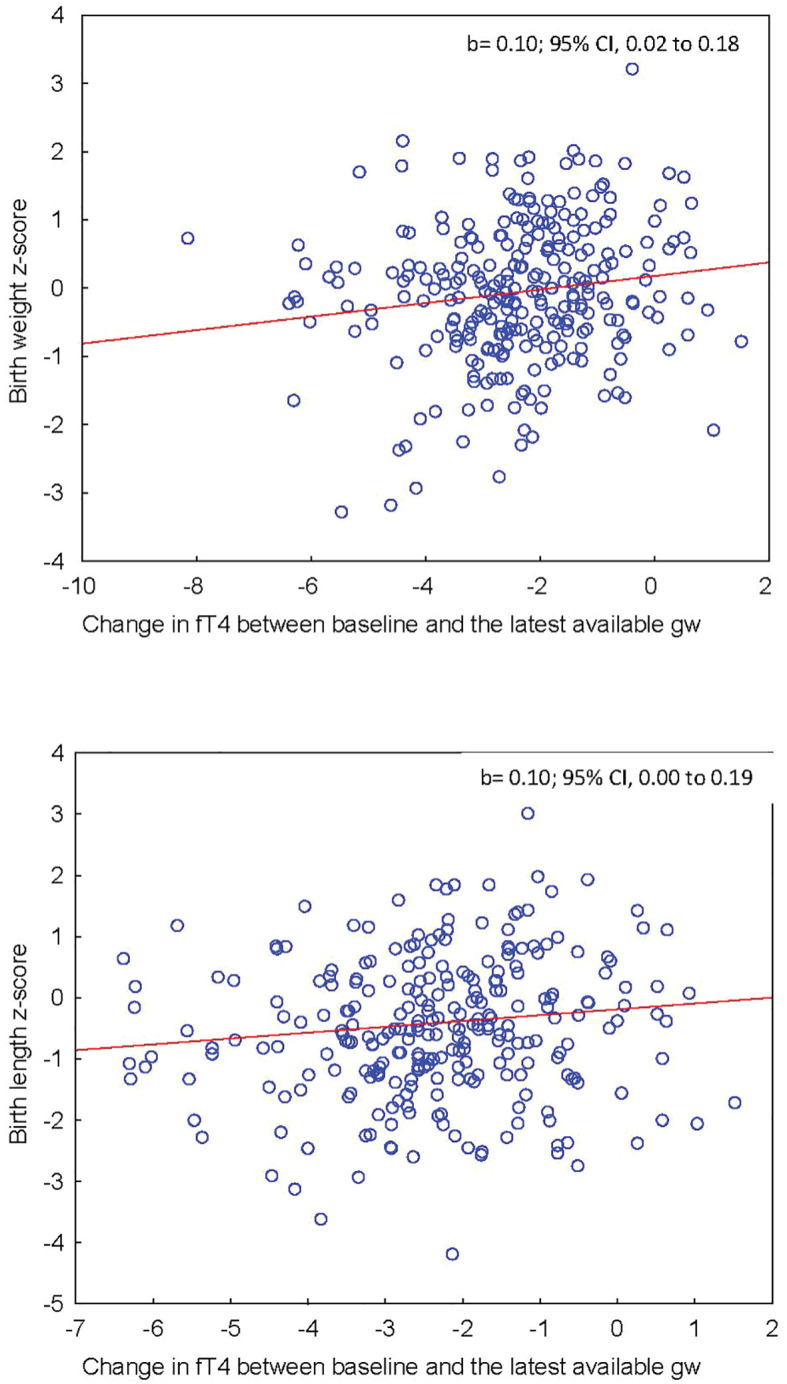
(Upper panel) Association between maternal ΔfT4 during pregnancy and offspring’s z-score birthweight adjusted for BMI. (Lower panel) Association between maternal ΔfT4 during pregnancy and offspring’s z-score birth length.

The associations between maternal thyroid parameters and offspring anthropometrics are shown in **Table 2**. Maternal fT4 at baseline tended to be negatively associated with z-score birthweight (0.07 SD lower birthweight for 1 pmol/l higher fT4, 95% CI, 0.00 to 0.14) and was significantly associated with birth length (0.09 SD shorter birth length for 1 pmol/L higher fT4; 95% CI, 0.00 to 0.17) (**Figure 3**). The change in fT4 during pregnancy (ΔfT4 latest available time-point - baseline) was positively associated with both z-score birthweight (0.10 SD higher birth weight for 1 pmol/L higher ΔfT4, 95% CI, 0.02 to 0.18) and z-score birth length (0.10 SD longer birth length for 1 pmol/L higher ΔfT4; 95% CI, 0.00 to 0.19) (**Figure 4**) meaning that a greater decrease in maternal fT4 during pregnancy was associated with a lower birthweight and shorter birth length. The aforementioned associations were observed independently of treatment group.

**Table 2:** Standard deviations in birthweight, birth length and birth head circumference z scores per 1 mIU/L increase in TSH and 1 pmol/L increase in fT4.

Multivariable Associations Between Thyroid Function Parameters and Birthweight z Scores by Study Visit, adjusted for BMI								
	Baseline (gw5–12)		Gw19		Gw32		Gw36	
Hormones	b (95% CI)	*p* value	b (95% CI)	*p* value	b (95% CI)	*p* value	b (95% CI)	*p* value
TSH	-0.01 (-0.14; 0.11)	0.827	-0.02 (-0.14; 0.10)	0.743	0.04 (-0.10; 0.17)	0.592	-0.02 (-0.17; 0.12)	0.753
fT4	-0.07 (-0.14; -0.00)	0.057	-0.05 (-0.13; 0.04)	0.271	-0.07 (-0.17; 0.02)	0.132	0.02 (-0.07; 0.11)	0.637
ΔTSH	0.03 (-0.12; 0.18) p=0.687							
ΔfT4	**0.10 (0.02; 0.18) p=0.017**							

Multivariable Associations Between Thyroid Function Parameters and Birth Length z Scores by Study Visit								
	Baseline (gw5–12)		Gw19		Gw32		Gw36	
Hormones	b (95% CI)	*p* value	b (95% CI)	*p* value	b (95% CI)	*p* value	b (95% CI)	*p* value
TSH	-0.02 (-0.18; 0.13)	0.756	P -0.24 (-0.48; 0.00)	0.053	0.01 (-0.16; 0.17)	0.951	**P -0.29 (-0.56; -0.03)**	0.032
			M 0.08 (-0.16; 0.32) *^1^	0.497			M 0.10 (-0.11; 0.30) *^2^	0.36
fT4	**-0.09 (-0.17; -0.00)**	**0.048**	-0.02 (-0.12; 0.07)	0.651	-0.08 (-0.19; 0.03)	0.15	-0.04 (-0.13; 0.06)	0.45
ΔTSH	-0.05 (-0.19; 0.10) p=0.540							
ΔfT4	**0.10 (0.00; 0.19) p=0.047**							

Multivariable Associations Between Thyroid Function Parameters and Birth Head Circumference z Scores by Study Visit, adjusted for BMI								
	Baseline (gw5–12)		Gw19		Gw32		Gw36	
Hormones	b (95% CI)	*p* value	b (95% CI)	*p* value	b (95% CI)	*p* value	b (95% CI)	*p* value
TSH	-0.07 (-0.20; 0.06)	0.306	-0.01 (-0.13; 0.12)	0.893	0.04 (-0.11; 0.18)	0.608	-0.03 (-0.18; 0.11)	0.658
fT4	-0.03 (-0.10; 0.05)	0.436	-0.02 (-0.11; 0.06)	0.569	-0.02 (-0.12; 0.07)	0.626	0.04 (-0.06; 0.13)	0.439
ΔTSH	0.07 (-0.09; 0.22) p=0.380							
ΔfT4	0.05 (-0.03; 0.13) p=0.227							

P, placebo group.

M, metformin group.

*^1^p value for interaction = 0.064.

*^2^p value for interaction = 0.024.

The bold values represent statistically significant results.

Maternal TSH was not associated with the offspring’s birth anthropometrics at any time-point during pregnancy, except for a negative association between TSH at gw 36 and z-score birth length (0.29 SD shorter birth length for 1 mIU/L higher TSH; 95% CI, 0.03 to 0.56) observed in the placebo group only (p-value for interaction between treatment groups: 0.024, non-statistically significant association for metformin group). Furthermore, there was no association between the change in TSH during pregnancy (ΔTSH latest available time-point - baseline) and birth anthropometrics.

**Table 3** shows the associations between maternal thyroid parameters and the outcomes LGA and SGA. Maternal fT4 at all time-points was not associated with LGA or SGA infants. Furthermore, ΔfT4 during pregnancy (latest available time-point - baseline) was not associated with the outcome of LGA. However, we found an association between ΔfT4 and SGA in the metformin group only (OR 0.58 per 1 pmol/L, 95% CI; 0.40 to 0.84), i.e. the odds for SGA decreased by 42% for every 1 pmol/L higher ΔfT4 during pregnancy when the mother was treated with metformin (p-value for interaction between treatment groups: 0.024, non-statistically significant association for placebo group).

**Table 3:** Associations between maternal TSH and fT4 and the outcomes LGA and SGA (OR per 1 mIU/L increase in TSH/ΔTSH and 1 pmol/L increase in fT4/ΔfT4).

Associations Between Maternal Thyroid Function Parameters and Odds of LGA Infant								
	Baseline (gw5–12)		Gw19		Gw32		Gw36	
Hormones	OR (95% CI)	*p* value	OR (95% CI)	*p* value	OR (95% CI)	*p* value	OR (95% CI)	*p* value
TSH	**0.47 (0.26–0.85)**	**0.012**	**0.58 (0.35–0.98)**	**0.042**	0.80 (0.47–1.36)	0.415	0.77 (0.48–1.24)	0.29
fT4	0.92 (0.73–1.17)	0.514	0.99 (0.77–1.28)	0.96	0.91 (0.67–1.25)	0.579	1.02 (0.77–1.34)	0.903
ΔTSH	**1.99 (1.10–3.61), p=0.023**							
ΔfT4	1.25 (0.94–1.65), p=0.122							

Associations Between Maternal Thyroid Function Parameters and Odds of SGA Infant								
	Baseline (gw5–12)		Gw19		Gw32		Gw36	
Hormones	OR (95% CI)	*p* value	OR (95% CI)	*p* value	OR (95% CI)	*p* value	OR (95% CI)	*p* value
TSH	P 0.93 (0.51–1.68)	0.811	0.70 (0.43–1.29)	0.142	0.86 (0.54–1.36)	0.516	0.85 (0.53–1.37)	0.507
	Μ 0.32 (0.14–0.71)^*1^	**0.005**						
fT4	1.08 (0.90–1.29)	0.407	1.10 (0.87–1.39)	0.414	1.05 (0.80–1.39)	0.725	0.93 (0.71–1.23)	0.623
ΔTSH	1.28 (0.80–2.07), p=0.307							
ΔfT4	P 1.02 (0.77–1.34), p=0.914							
	M 0.58 (0.40–0.84), p=0.004 *^2^							

*^1^p value for interaction= 0.035.

*^2^p value for interaction= 0.024.

M, metformin group.

P, placebo group.

The bold values represent statistically significant results.

Baseline TSH was inversely associated with LGA infant (OR 0.47 per 1 mIU/L, 95% CI, 0.26 to 0.85), i.e the odds for LGA decreased by 53% for every 1 mIU/L higher TSH at baseline. Moreover, TSH at gw19 was also inversely associated with LGA infant (OR 0.58 per 1 mIU/L, 95% CI, 0.35 to 0.98), meaning that the odds for LGA decreased by 42% for 1 mIU/L higher TSH at gw19. Furthermore, ΔTSH during pregnancy (latest available time-point - baseline) was significantly associated with increased odds for LGA (OR 1.99 per 1 mIU/L, 95% CI, 1.10 to 3.61, i.e the odds for LGA was almost 2-fold for 1 mIU/L greater increase in TSH during pregnancy. The association between TSH at baseline and SGA differed between the treatment groups (p-value for interaction 0.035) and TSH at baseline was significantly associated with SGA in the metformin group only (OR 0.32 per 1 mIU/L, 95% CI, 0.14 to 0.71), which means that the odds for SGA was 68% lower for 1 mIU/L higher TSH at baseline when the mother was treated with metformin. There was no evidence of association in the placebo group.

No statistically significant difference was observed between TPO-positive and TPO-negative groups regarding z-scores (birthweight, length, head circumference) and LGA/SGA (data not shown).

The effects of metformin on birth anthropometrics of the offspring of women with PCOS were described by Nilsen et al. (2023) (26). Since our population is slightly different from the population in the aforementioned study, we performed new analyses regarding the effect of metformin versus placebo on birth anthropometrics (**Table 4**), which confirmed that head circumference was larger in the metformin group than in the placebo group (p=0.003 and p-value for z-score=0.043). However, no associations were observed between maternal fT4 and TSH during pregnancy and z-score head circumference of the newborns. The prevalence of SGA and LGA did not differ between the treatment groups, p=0.556 and p=0.808 respectively.

**Table 4:** Anthropometrics at birth of offspring born to mothers with PCOS exposed to metformin (n=153) or placebo (n=156).

Variable	(n)	Total	(n)	Metformin	(n)	Placebo	p-value
Gestational age (weeks)	309	39.1 ± 2.0	153	39.3 ± 1.6	156	38.9 ± 2.3	0.533
Weight							
grams	309	3529 ± 600	153	3555 ± 551	156	3504 ± 647	0.455
z-score	309	-0.05 (-0.17; 0.06)	153	-0.10 (-0.27; 0.08)	156	-0.01 (-0.17; 0.14)	0.474
Length							
cm	305	50 ± 3	152	50 ± 2	153	50 ± 3	0.901
z-score	305	-0.52 *(-0.66; -0.38)*	152	-0.63 *(-0.83; -0.44)*	153	-0.41*(-0.60; -0.22)*	0.101
Head circumference							
cm	306	35 ± 2	152	36 ± 2	154	35 ± 2	***0.003***
z-score	306	0.19 *(0.06; 0.31)*	152	0.31 *(0.13; 0.49)*	154	0.06 (-0.10; 0.22)	***0.043***
SGA n (%)		37 (12)		20 (13)		17 (11)	0.556
LGA n (%)		33 (11)		17 (11)		16 (10)	0.808

Values expressed with a plus/minus sign are the mean ± standard deviation.

Values in parentheses are 95% CIs.

Students t- test was performed. T-test with separate variance estimates was used for birthweight in grams because of non-homogenous variances.

SGA (small for gestational age): weight z-score< -1.28.

LGA (large for gestational age): weight z-score >1.28.

The bold values represent statistically significant results.

## Discussion

This is the first study exploring associations between maternal thyroid function and birth anthropometrics/adverse growth outcomes of the offspring in women with PCOS. We found that maternal fT4 in early pregnancy (most of the values within the normal range) was inversely associated with offspring birthweight and birth length. Moreover, a greater decrease in maternal fT4 during pregnancy was associated with a lower birthweight and shorter birth length. These results were independent of metformin treatment. Furthermore, a higher maternal TSH at baseline and gw19 (most of the values within the normal range) was associated with a lower risk for LGA, while a greater increase in TSH during pregnancy was associated with a higher risk for LGA. In the metformin group only, a higher TSH at baseline, and a smaller decrease in fT4 during pregnancy were associated with a lower risk of SGA.

Our findings of associations between thyroid hormone levels and offspring anthropometrics in women with PCOS, confirm results of previous studies in non-PCOS women. An inverse association between fT4 over the course of pregnancy and birth weight *z*-scores was reported by Johns et al. (2018) in a nested case-control study. In accordance with our study, fT4 at first trimester (median 10 weeks of gestation) showed the strongest association with birthweight (13). Similar negative associations between maternal fT4 in early pregnancy and offspring birthweight were described in the Generation R cohort (2013) and by Vrijkotte et al. (2017) among a community-based cohort of pregnant women in Amsterdam (14, 16). In the FaSTER trial, an observational cohort study (2014), the lowest median offspring birth weight occurred among non-PCOS women in the highest fT4 quintile during the second pregnancy trimester (12). Furthermore, in a cohort study, Shields et al. (2011), reported a negative association between maternal fT4 in the third trimester and birthweight, birth length and head circumference of the offspring, respectively (30).

During a normal pregnancy, the concentration of fT4 increases from conception to just before the end of the first trimester when the formation of the placenta is completed, and then gradually decreases until delivery (**Figure 1**). This gradual decrease in fT4 follows the parallel increasing degradation of T4 by type 3 deiodinase (D3), which is highly expressed in the placenta, pregnant uterus, and fetal tissues (1, 31). A greater fT4 decrease during pregnancy, associated with lower birthweight and birth length according to our findings, could possibly be a proxy for a metabolically altered milieu which affects fetal growth. A potential mechanism for this effect could involve derangements in the expression and activity of deiodinases, leading to changes in maternal (and fetal/neonatal) thyroid status, as proposed in metabolic imbalance such as gestational diabetes (32). Studies in rats have shown that hypothyroidism impacts fetal weight by adversely affecting angiogenesis and inducing apoptosis in the placenta (33, 34) and this might be a possible mechanism connecting the greater fT4 decrease with lower birth weight/length in the offspring.

The present study shows no evidence of associations between maternal TSH during pregnancy and offspring’s anthropometry, apart from an inverse association between TSH at gw36 and birth length for the placebo group only. In a cohort study by Alvarez-Pedrerol et al. (2009) in non-PCOS pregnant women, higher TSH levels at the first trimester were associated with a higher risk of low birth weight or SGA (7). Nishioka et al. (2015) reported that among normal pregnant women, the change in TSH between gestational weeks 12 and 36 was inversely correlated with birthweight, concluding that an increase in maternal TSH concentration between the first and third trimesters is an independent determinant of birth weight (17). In the same study, the median TSH concentration increased during pregnancy 1.63-fold in the normal group and 4.3-fold in the group of low birthweight newborns. In our whole cohort, the increase in TSH during pregnancy was only 1.48-fold, which may explain the lack of impact on birthweight. Finally, our results are in accordance with those published by Orito et al. (2009), who found no correlation between maternal TSH in early pregnancy of healthy Japanese women and birth parameters (weight, height, head circumference) of neonates (35).

Although we found clear associations between fT4 and ΔfT4 and birth parameters (weight and length), the associations with more crude outcomes such as LGA and SGA may possibly be more clinically relevant. In an observational study from China, hypothyroidism was associated with an increased risk of SGA, while hyperthyroxinaemia was associated with a decreased risk of LGA (36). However, we found that a higher TSH in the first trimester and by mid-gestation among PCOS women was associated with 40–50% reduced risk for LGA, while every unit increase in TSH throughout the pregnancy doubled the odds for LGA.

In the present study, we found no associations between maternal thyroid status and head circumference z-score of the newborns at any time point during pregnancy. Thus, the larger head circumference of the offspring in the metformin group, which has also been reported previously (26) does not seem to be mediated by thyroid hormones.

Most of the associations in the current study did not differ between the metformin and placebo groups, as the interaction of TSH/fT4 with treatment was not statistically significant. The inverse associations of ΔfT4 during pregnancy and baseline TSH with SGA were statistically significant in the metformin group only. Thus, we did not notice a consistent pattern that could support any convincing modifying effect of metformin on the observed associations.

The strengths of our study include the well-characterized population of women with PCOS and the prospective and longitudinal data sampling, allowing us to assess the maternal thyroid status through pregnancy. Even though our study is a *post-hoc* analysis from two trials randomizing the participants into metformin and placebo groups, the study design allowed us to collect information from all the participants combined, taking into consideration their group division in the statistical model. One may consider the lack of control group as a limitation, however this was beyond the scope of our study. Moreover, since the original RCTs were not designed to test the associations investigated in the current study, we acknowledge that our results would need confirmation in a more adequately powered study. Furthermore, the method used to measure fT4 in our study (chemiluminescent enzyme immunoassay) may overestimate the fall in fT4 during the 2^nd^ and 3^rd^ trimester compared to the reference methods (equilibrium dialysis, gas chromatography/mass spectrometry) (37), which may have impacted our results and their interpretation. Furthermore, the study sample size did not allow for stratifying analysis of outcomes by sex, since sexual dimorphism may affect the relationship between maternal thyroid metabolism and fetal growth (16). However, the outcomes birthweight, birth length and head circumference as presented as z-scores, which are per definition adjusted for sex and gestational age.

In conclusion, we have demonstrated that a relatively higher fT4 within the normal range in early pregnancy, as well as a greater decline of fT4 during pregnancy are associated with lower birthweight and birth length of the offspring of women with PCOS. Moreover, a relatively higher TSH within the normal range by mid-gestation is associated with a reduced risk for delivery of LGA, and so is a smaller TSH increase throughout pregnancy. These associations may reflect an interplay between maternal thyroid function and the placental milieu of importance for birth anthropometrics of the offspring and the risk for adverse growth outcomes. However, future studies are needed to confirm our results and to elucidate possible underlying mechanisms to these associations.

## Data availability statement

The original contributions presented in the study are included in the article/**Supplementary Material**. Further inquiries can be directed to the corresponding author.

## Ethics statement

The studies involving humans were approved by The Committee for Medical Research Ethics of Health Region IV, Norway. The studies were conducted in accordance with the local legislation and institutional requirements. The participants provided their written informed consent to participate in this study.

## Author contributions

AT: Writing – original draft, Writing – review & editing. MA: Writing – review & editing. JC: Writing – review & editing. BÅ: Writing – review & editing. DU: Writing – review & editing. AH: Writing – original draft, Writing – review & editing. EV: Writing – original draft, Writing – review & editing.

## References

[1] Korevaar TIM Medici M Visser TJ Peeters RP. Thyroid disease in pregnancy: new insights in diagnosis and clinical management. Nat Rev Endocrinol. (2017) 13:610–22. doi: 10.1038/nrendo.2017.93 28776582

[2] van den Boogaard E Vissenberg R Land JA van Wely M van der Post JA Goddijn M. Significance of (sub)clinical thyroid dysfunction and thyroid autoimmunity before conception and in early pregnancy: a systematic review. Hum Reprod Update. (2011) 17:605–19. doi: 10.1093/humupd/dmr024 21622978

[3] Mahadik K Choudhary P Roy PK. Study of thyroid function in pregnancy, its feto-maternal outcome; a prospective observational study. BMC Pregnancy Childbirth. (2020) 20:769. doi: 10.1186/s12884-020-03448-z 33302910 PMC7726876

[4] Mannisto T Mendola P Grewal J Xie Y Chen Z Laughon SK. Thyroid diseases and adverse pregnancy outcomes in a contemporary US cohort. J Clin Endocrinol Metab. (2013) 98:2725–33. doi: 10.1210/jc.2012-4233 PMC3701274 23744409

[5] Männistö T Vääräsmäki M Pouta A Hartikainen AL Ruokonen A Surcel HM. Perinatal outcome of children born to mothers with thyroid dysfunction or antibodies: a prospective population-based cohort study. J Clin Endocrinol Metab. (2009) 94:772–9. doi: 10.1210/jc.2008-1520 19106271

[6] Thangaratinam S Tan A Knox E Kilby MD Franklyn J Coomarasamy A. Association between thyroid autoantibodies and miscarriage and preterm birth: meta-analysis of evidence. BMJ (Clinical Res ed). (2011) 342:d2616. doi: 10.1136/bmj.d2616 PMC3089879 21558126

[7] Alvarez-Pedrerol M Guxens M Mendez M Canet Y Martorell R Espada M. Iodine levels and thyroid hormones in healthy pregnant women and birth weight of their offspring. Eur J Endocrinol / Eur Fed Endocrine Societies. (2009) 160:423–9. doi: 10.1530/eje-08-0716 19114540

[8] Chen LM Du WJ Dai J Zhang Q Si GX Yang H. Effects of subclinical hypothyroidism on maternal and perinatal outcomes during pregnancy: a single-center cohort study of a Chinese population. PloS One. (2014) 9:e109364. doi: 10.1371/journal.pone.0109364 25353960 PMC4212915

[9] Karakosta P Alegakis D Georgiou V Roumeliotaki T Fthenou E Vassilaki M. Thyroid dysfunction and autoantibodies in early pregnancy are associated with increased risk of gestational diabetes and adverse birth outcomes. J Clin Endocrinol Metab. (2012) 97:4464–72. doi: 10.1210/jc.2012-2540 23015651

[10] Su PY Huang K Hao JH Xu YQ Yan SQ Li T. Maternal thyroid function in the first twenty weeks of pregnancy and subsequent fetal and infant development: a prospective population-based cohort study in China. J Clin Endocrinol Metab. (2011) 96:3234–41. doi: 10.1210/jc.2011-0274 21832110

[11] Cleary-Goldman J Malone FD Lambert-Messerlian G Sullivan L Canick J Porter TF. Maternal thyroid hypofunction and pregnancy outcome. Obstetrics Gynecology. (2008) 112:85–92. doi: 10.1097/AOG.0b013e3181788dd7 18591312 PMC4949950

[12] Haddow JE Craig WY Neveux LM Haddow HR Palomaki GE Lambert-Messerlian G. Implications of High Free Thyroxine (FT4) concentrations in euthyroid pregnancies: the FaSTER trial. J Clin Endocrinol Metab. (2014) 99:2038–44. doi: 10.1210/jc.2014-1053 PMC4037729 24606107

[13] Johns LE Ferguson KK Cantonwine DE Mukherjee B Meeker JD McElrath TF. Subclinical changes in maternal thyroid function parameters in pregnancy and fetal growth. J Clin Endocrinol Metab. (2018) 103:1349–58. doi: 10.1210/jc.2017-01698 PMC6018657 29293986

[14] Medici M Timmermans S Visser W de Muinck Keizer-Schrama SM Jaddoe VW Hofman A. Maternal thyroid hormone parameters during early pregnancy and birth weight: the Generation R Study. J Clin Endocrinol Metab. (2013) 98:59–66. doi: 10.1210/jc.2012-2420 23150694

[15] Nazeri P Shab-Bidar S Pearce EN Shariat M. Do maternal urinary iodine concentration or thyroid hormones within the normal range during pregnancy affect growth parameters at birth? A systematic review and meta-analysis. Nutr Rev. (2020) 78:747–63. doi: 10.1093/nutrit/nuz105 31923312

[16] Vrijkotte TG Hrudey EJ Twickler MB. Early maternal thyroid function during gestation is associated with fetal growth, particularly in male newborns. J Clin Endocrinol Metab. (2017) 102:1059–66. doi: 10.1210/jc.2016-3452 28359096

[17] Nishioka E Hirayama S Ueno T Matsukawa T Vigeh M Yokoyama K. Relationship between maternal thyroid-stimulating hormone (TSH) elevation during pregnancy and low birth weight: a longitudinal study of apparently healthy urban Japanese women at very low risk. Early Hum Dev. (2015) 91:181–5. doi: 10.1016/j.earlhumdev.2014.12.014 25676185

[18] Yildiz BO Bozdag G Yapici Z Esinler I Yarali H. Prevalence, phenotype and cardiometabolic risk of polycystic ovary syndrome under different diagnostic criteria. Hum Reprod (Oxford England). (2012) 27:3067–73. doi: 10.1093/humrep/des232 22777527

[19] Azziz R Carmina E Chen Z Dunaif A Laven JS Legro RS. Polycystic ovary syndrome. Nat Rev Dis Primers. (2016) 2:16057. doi: 10.1038/nrdp.2016.57 27510637

[20] DeUgarte CM Bartolucci AA Azziz R. Prevalence of insulin resistance in the polycystic ovary syndrome using the homeostasis model assessment. Fertil Steril. (2005) 83:1454–60. doi: 10.1016/j.fertnstert.2004.11.070 15866584

[21] Ding X Yang L Wang J Tang R Chen Q Pan J. Subclinical hypothyroidism in polycystic ovary syndrome: A systematic review and meta-analysis. Front Endocrinol. (2018) 9:700. doi: 10.3389/fendo.2018.00700 PMC6277795 30542323

[22] Romitti M Fabris VC Ziegelmann PK Maia AL Spritzer PM. Association between PCOS and autoimmune thyroid disease: a systematic review and meta-analysis. Endocrine Connections. (2018) 7:1158–67. doi: 10.1530/ec-18-0309 PMC6215798 30352422

[23] Teede HJ Tay CT Laven JJE Dokras A Moran LJ Piltonen TT. Recommendations from the 2023 international evidence-based guideline for the assessment and management of polycystic ovary syndrome. J Clin Endocrinol Metab. (2023) 108: 2447–69. doi: 10.1210/clinem/dgad463 37580314 PMC10505534

[24] Lovvik TS Carlsen SM Salvesen O Steffensen B Bixo M Gomez-Real F. Use of metformin to treat pregnant women with polycystic ovary syndrome (PregMet2): a randomised, double-blind, placebo-controlled trial. Lancet Diabetes Endocrinol. (2019) 7:256–66. doi: 10.1016/s2213-8587(19)30002-6 30792154

[25] Trouva A Alvarsson M Calissendorff J Åsvold BO Vanky E Hirschberg AL. Thyroid status during pregnancy in women with polycystic ovary syndrome and the effect of metformin. Front Endocrinol. (2022) 13:772801. doi: 10.3389/fendo.2022.772801 PMC8898827 35265033

[26] Nilsen G Simpson MR Hanem LGE Løvvik TS Ødegård R Stokkeland LMT. Anthropometrics of neonates born to mothers with PCOS with metformin or placebo exposure in *utero* . Acta Obstet Gynecol Scand. (2023) 103: 176–87. doi: 10.1111/aogs.14637 37488743 PMC10755130

[27] Vanky E Salvesen KA Heimstad R Fougner KJ Romundstad P Carlsen SM. Metformin reduces pregnancy complications without affecting androgen levels in pregnant polycystic ovary syndrome women: results of a randomized study. Hum Reprod (Oxford England). (2004) 19:1734–40. doi: 10.1093/humrep/deh347 15178665

[28] Vanky E Stridsklev S Heimstad R Romundstad P Skogoy K Kleggetveit O. Metformin versus placebo from first trimester to delivery in polycystic ovary syndrome: a randomized, controlled multicenter study. J Clin Endocrinol Metab. (2010) 95:E448–55. doi: 10.1210/jc.2010-0853 20926533

[29] Niklasson A Albertsson-Wikland K. Continuous growth reference from 24th week of gestation to 24 months by gender. BMC Pediatr. (2008) 8:8. doi: 10.1186/1471-2431-8-8 18307822 PMC2294116

[30] Shields BM Knight BA Hill A Hattersley AT Vaidya B. Fetal thyroid hormone level at birth is associated with fetal growth. J Clin Endocrinol Metab. (2011) 96:E934–8. doi: 10.1210/jc.2010-2814 PMC3100744 21411545

[31] Huang SA Dorfman DM Genest DR Salvatore D Larsen PR. Type 3 iodothyronine deiodinase is highly expressed in the human uteroplacental unit and in fetal epithelium. J Clin Endocrinol Metab. (2003) 88:1384–8. doi: 10.1210/jc.2002-021291 12629133

[32] Gutiérrez-Vega S Armella A Mennickent D Loyola M Covarrubias A Ortega-Contreras B. High levels of maternal total tri-iodothyronine, and low levels of fetal free L-thyroxine and total tri-iodothyronine, are associated with altered deiodinase expression and activity in placenta with gestational diabetes mellitus. PloS One. (2020) 15:e0242743. doi: 10.1371/journal.pone.0242743 33232364 PMC7685482

[33] Silva JF Vidigal PN Galvão DD Boeloni JN Nunes PP Ocarino NM. Fetal growth restriction in hypothyroidism is associated with changes in proliferative activity, apoptosis and vascularisation of the placenta. Reprod Fertil Dev. (2012) 24:923–31. doi: 10.1071/rd11219 22935153

[34] Silva JF Ocarino NM Serakides R. Placental angiogenic and hormonal factors are affected by thyroid hormones in rats. Pathol Res Pract. (2015) 211:226–34. doi: 10.1016/j.prp.2014.11.003 25499719

[35] Orito Y Oku H Kubota S Amino N Shimogaki K Hata M. Thyroid function in early pregnancy in Japanese healthy women: relation to urinary iodine excretion, emesis, and fetal and child development. J Clin Endocrinol Metab. (2009) 94:1683–8. doi: 10.1210/jc.2008-2111 19258403

[36] Yuan X Wang J Gao Y Wang H Yu B. Impact of maternal thyroid hormone in late pregnancy on adverse birth outcomes: A retrospective cohort study in China. Endocrine J. (2021) 68:317–28. doi: 10.1507/endocrj.EJ20-0377 33115985

[37] Lee RH Spencer CA Mestman JH Miller EA Petrovic I Braverman LE. Free T4 immunoassays are flawed during pregnancy. Am J Obstetrics Gynecology. (2009) 200:260.e1–6. doi: 10.1016/j.ajog.2008.10.042 19114271

