# Supplementary material for: Maternal thyroid function and offspring birth anthropometrics in women with polycystic ovary syndrome
Source: Front Endocrinol (Lausanne). 2024 May 29;15:1388473. doi: 10.3389/fendo.2024.1388473 (PMC11167103; doi:10.3389/fendo.2024.1388473)
Supplement: Supplementary file 1 [file Table_1.docx]

**Supplementary material**

All participants in the Pilot and PregMet 1 studies met the Rotterdam criteria (retrospectively evaluated in the Pilot study, as it was conducted prior to the establishment of the Rotterdam criteria), additional inclusion criteria were: age 18-45 years, gestational age between weeks 5-12, and a singleton viable fetus shown on ultrasonography. Exclusion criteria were alanine aminotransferase higher than 90 nmol/L, serum creatinine higher than 139 mmol/L, known alcohol abuse, previously diagnosed diabetes mellitus or fasting serum glucose higher than at inclusion, treatment with oral glucocorticoids, or use of drugs known to interfere with metformin. Randomization to metformin or placebo was stratified according to metformin use at conception. All participants were assigned in a double-blind manner either to metformin or placebo group. In both studies the participants received written and individual verbal counselling on diet and lifestyle at inclusion. Thereafter, treatment with metformin or identically coated placebo tablets was initiated for the rest of the study period. The participants received 850 mg (Pilot study) or 1000 mg (PregMet 1 study) metformin or placebo twice daily. Women who used metformin at conception had a “wash-out” period of ≥7 days before inclusion. All the participants received a 1mg tablet of folate daily, and one daily multivitamin tablet containing: vitamin A 800 mg, vitamin B1 1.4 mg, vitamin B2 1.6 mg, vitamin B6 2 mg, vitamin B12 1 mg, folic acid 200 mg, niacin 18 mg, pantotenic acid 6 mg, vitamin C 60 mg, vitamin D 5 mg, vitamin E 10 mg, Fe2+ 14 mg, Zn+ 15 mg, Cu2+ 2 mg, iodine 150 mg, Mn2+ 2.5 mg, Cr+ 50 mg, and Se+ 50 mg (Vitaplexw; Alpharma AS, Norway). Study medication was stopped at delivery. Fasting blood samples were drawn at inclusion (gestational week (gw) 5-12), gw 19 ± 1, gw 32 ± 1, and at gw 36 ± 1.

An intake of more than 85% of the prescribed tablets was self-reported by 80% of the participants, which were thus considered to have good/acceptable compliance.
