# Supplementary material for: Maternal thyroid function and offspring birth anthropometrics in women with polycystic ovary syndrome
Source: Front Endocrinol (Lausanne). 2024 May 29;15:1388473. doi: 10.3389/fendo.2024.1388473 (PMC11167103; doi:10.3389/fendo.2024.1388473)
Supplement: Supplementary file 2 [file Table_2.docx]

|  | Total | Metformin | Placebo | p-value |
| --- | --- | --- | --- | --- |
| TSH _incl_ | **1.26** (0.82-1.93) | **1.29** (0.82-1.99) | **1.23** (0.83-1.81) | *0.326* |
| TSH _gw19_ | **1.75** (1.20-2.27) | **1.77** (1.22-2.28) | **1.75** (1.20-2.25) | *0.945* |
| TSH _gw32_ | **1.81** (1.24-2.24) | **1.89** (1.27-2.32) | **1.69** (1.20-2.06) | ***0.034*** |
| TSH _gw36_ | **1.87** (1.28-2.41) | **1.96** (1.27-2.52) | **1.77** (1.32-2.28) | *0.167* |
|  |  |  |  |  |
| fT4 _incl_ | **14.16** (13.13-15.19) | **14.16** (13.13-15.19) | **14.16** (13.26-15.19) | *0.727* |
| fT4 _gw19_ | **12.47** (11.66-13.51) | **12.87** (11.93-13.77) | **12.31** (11.36-13.26) | ***0.001*** |
| fT4 _gw32_ | **11.78** (10.88-12.64) | **11.92** (11.18-12.87) | **11.61** (10.64-12.42) | ***0.006*** |
| fT4 _gw36_ | **11.92** (10.86-13.00) | **12.21** (11.07-13.00) | **11.71** (10.68-12.63) | ***0.025*** |

Abbreviations: TSH= thyroid stimulating hormone, fT4= free thyroxine, gw= gestational week

Values expressed as median and 25^th^-75^th^ percentiles

(TSH was significantly higher in the metformin group at gw 32 (p<0.05) whereas fT4 was significantly higher (although within the reference range) in the metformin group at gw 19 (p<0.01), gw32 (p<0.01) and gw36 (p<0.05).)
